# Integrated Analysis of Copy Number Variation, Microsatellite Instability, and Tumor Mutation Burden Identifies an 11-Gene Signature Predicting Survival in Breast Cancer

**DOI:** 10.3389/fcell.2021.721505

**Published:** 2021-09-28

**Authors:** Xin Jin, Junfeng Yan, Chuanzhi Chen, Yi Chen, Wen-Kuan Huang

**Affiliations:** ^1^Department of Breast Surgery, Zhuji Affiliated Hospital of Shaoxing University, Zhuji, China; ^2^Department of Surgical Oncology, The First Affiliated Hospital, School of Medicine, Zhejiang University, Hangzhou, China; ^3^Department of Oncology-Pathology, Karolinska Institute, Stockholm, Sweden; ^4^Division of Hematology-Oncology, Department of Internal Medicine, Chang Gung Memorial Hospital at Linkou, Chang Gung University College of Medicine, Taoyuan, Taiwan

**Keywords:** breast cancer, prognostic signature, genetic variants, copy number variation, microsatellite instability, tumor mutation burden

## Abstract

Genetic variants such as copy number variation (CNV), microsatellite instability (MSI), and tumor mutation burden (TMB) have been reported to associate with the immune microenvironment and prognosis of patients with breast cancer. In this study, we performed an integrated analysis of CNV, MSI, and TMB data obtained from The Cancer Genome Atlas, thereby generating two genetic variants-related subgroups. We characterized the differences between the two subgroups in terms of prognosis, MSI burden, TMB, CNV, mutation landscape, and immune landscape. We found that cluster 2 was marked by a worse prognosis and lower TMB. According to these groupings, we identified 130 differentially expressed genes, which were subjected to univariate and least absolute shrinkage and selection operator-penalized multivariate modeling. Consequently, we constructed an 11-gene signature risk model called the genomic variation-related prognostic risk model (GVRM). Using ROC analysis and a calibration plot, we estimated the prognostic prediction of this GVRM. We confirmed the predictive efficiency of this GVRM by validating it in another independent International Cancer Genome Consortium cohort. Our results conclude that an 11-gene signature developed by integrated analysis of CNV, MSI, and TMB has a high potential to predict breast cancer prognosis, which provided a strong rationale for further investigating molecular mechanisms and guiding clinical decision-making in breast cancer.

## Introduction

Breast cancer is the most commonly diagnosed cancer and the second leading cause of cancer-related deaths in women worldwide (DeSantis et al., 2019). While patients diagnosed with early-stage tumors have increased the 5-year survival rate to more than 90%, the 5-year survival rate for patients with metastatic diseases is reduced to ∼25% with currently available systemic treatment modalities including chemotherapy, endocrine therapy, and targeted biological therapy (Bonotto et al., 2014). The success of immune checkpoint blockade (ICB) in melanoma, non-small cell lung cancer, and other solid tumors has led to emerging enthusiasm for investigating immunotherapy to treat patients with breast cancer. Breast tumors have been generally considered immunologically cold, characterized by limited effector lymphocyte infiltration, and low mutation and neoantigen burden (Bates et al., 2018; Adams et al., 2019). Therefore, there is still an unmet need in identifying novel biomarkers for patients who might benefit from ICB therapy.

Some common genetic variants in cancer cells have been shown to associate with PD-1– and PD-L1–based immunotherapy (Cristescu et al., 2018). Pan-cancer analyses revealed that high copy number variation (CNV) was associated with increased proliferation signature and decreased immune infiltration signature (Taylor et al., 2018). Correspondingly, somatic CNV was reported to negatively correlate with response to CTLA-4 and PD-1/PD-L1 blockade (Davoli et al., 2017). High tumor mutation burden (TMB) has emerged as a biomarker of responsiveness to immunotherapy in several tumor types (Rizvi et al., 2015; Samstein et al., 2019). Microsatellite instability (MSI) was also recognized as a predictive marker for immunotherapy response (Le et al., 2015, 2017). MSI caused by deficiency of DNA mismatch repair genes is associated with accumulation of mutational load and neoantigen production, which may contribute to the better response of ICB. Of note, MSI and TMB were subsequently approved by the FDA as tissue-agnostic predictive biomarkers for pembrolizumab (Passaro et al., 2020). Moreover, CNV, TMB, and MSI were found to be associated with breast cancer prognosis (Horlings et al., 2010; Fusco et al., 2018; Thomas et al., 2018). Hence, integrated analysis of gene expression based on these three genetic variants may more accurately identify a gene signature model to predict the immune response and prognosis in breast cancer.

In this study, we performed an integrated clustering of somatic CNV, MSI, and TMB of 1,079 breast cancer samples from The Cancer Genome Atlas (TCGA) database, for which complete clinical parameters and prognosis are available. Consensus clustering based on k-means revealed two subgroups. We further explored their differences in survival, mutation pattern, TMB, CNV, immune cell infiltration, and potential immune response of ICB by the Tumor Immune Dysfunction and Exclusion (TIDE) algorithm. Next, we identified an 11-gene signature associated with prognosis based on the differentially expressed genes (DEGs) between the two subgroups using least absolute shrinkage and selection operator (LASSO) Cox regression. The prognostic impact of this genetic variants-related risk score was validated with external datasets. To predict survival for an individual patient with breast cancer, we developed a prognostic nomogram model by integrating the genetic variants-related risk score and clinicopathological features. The performance of the nomogram was evaluated by calibration curve and decision curve analysis.

## Materials and Methods

### Data Source

The Cancer Genome Atlas-BRCA gene expression data (*n* = 1,072), mutation data (*n* = 986), copy number data (*n* = 1,080), and corresponding clinical data (*n* = 1,079) were downloaded from the UCSC Xena^1^ website. The patients’ characteristics involved are shown in Supplementary Table 1. In this study, we investigated the transcriptional data in both counts and Fragments Per Kilobase of transcript per Million mapped reads (FPKM) values, raw counts data were used for differential expression analyses, while the gene expression units for downstream analyses were transformed with log2([FPKM] + 1). We selected significantly mutated genes derived by identification of driver gene studies and a molecular portrait of MSI across multiple cancers (Kandoth et al., 2013; Cortes-Ciriano et al., 2017). The BRCA-KR data from the International Cancer Genome Consortium (ICGC) was used as an independent validation cohort (*n* = 50).

### Microsatellite Instability Data Processing

Microsatellite instability was identified at the transcriptional level using the PreMSIm algorithm (Li et al., 2020) in R software for TCGA-BRCA data. Under the default parameters, PreMSIm first scanned the FPKM data (log2-transformed) to distinguish MSI-high (MSI-H) cancers from MSI-low/microsatellite stability (MSS) samples, leading to obtaining a binary matrix for MSI identification. In this MSI classification call, 1 represents MSI, and 0 represents MSS. Samples of quantitative MSI burden were obtained from previous work done by Cortes-Ciriano et al. (2017).

### Somatic Copy Number Alteration Data Processing

The somatic copy number alteration (SCNA) genome feature was defined as a repetitive region with a change in copy number determined by the algorithm GISTIC2.0 (Mermel et al., 2011). We determined the SCNA features and their binary status in each sample by using the SCNA processing method based on a previous study (Ciriello et al., 2013). The specific methods are illustrated as follows: We extracted the peak area of GISTIC results of all tumor types as SCNA features. For peak regions with the same gene, only one peak region was reserved. To determine copy number alteration events, we used the set of discrete copy number calls provided by GISTIC: homozygous deletion (−2); hemizygous deletion (−1); diploid (0); low-level gain (1); and high-level amplification (2). When more than half of the genes in the amplified or deleted peak region were high-level amplification (2) or homozygous deletion (−2), the copy number of the peak region is defined as changed. To obtain the SCNA by a sample binary matrix, we assigned a value to each SCNA feature of each sample where 1 represents feature changes, and 0 represents no feature changes, leading to an SCNA feature description matrix.

SCNA burden scores were computed using masked copy number segment data from the UCSC Xena website and defined as the sum of the log2-transformed copy-number ratio (tumor vs. normal) of genomic segments normalized by segment length.

### modified TMB Data Processing

modified TMB (mTMB) was defined as the total number of unique genes with mutations. Only seven types of mutations were considered in this study: Nonsense_Mutation, Nonstop_ Mutation, Missense_Mutation, Frame_Shift_del, Frame_Shift_Ins, Splice_Site, and Translation_Start_Site. For mTMB features, (1) after merging the MAF data of TCGA-BRCA, we extracted 1,399 most frequently mutated genes according to the cut-off of a certain gene mutation sample accounting for 1% of the total number of samples; (2) significantly mutated genes were obtained by identification of driver gene studies (Kandoth et al., 2013).

### Integrating of Genomic Variation Data

We constructed a total of 1,536 genome variant features, including 46 copy number amplifications, 21 copy number deletions, 1 MSI, and 1,468 genes after a series of data processing. We then characterized the SCNA, MSI, and mTMB features in each tumor sample in a binary manner to indicate whether genomic variations occurred in each tumor sample. This resulted in three binary features of the sample matrix constructed by SCNA, MSI, and mTMB, where 1 represents the presence of genomic alterations, and 0 represents no genomic alterations in the matrix. Here, the above three matrices were called genome variation feature description matrices.

### Sample Clustering Based on Genomic Features

We integrated the three genomic variation feature description matrices into one matrix initially. The columns and rows represent the sample and the corresponding genomic variation, respectively. We got a total of 961 valid samples. R package “iClusterPlus” (Mo et al., 2013) was used for integrative clustering analysis of multi-type genomic variations. Under the processing of default parameters, we tried different classification modes at *k* = 1–5, and finally *k* = 1, that is, cluster = 2 as the optimal classification result (Supplementary Figure 1). For the features selection, we used the quartile of the sum of the beta values of a certain feature in all samples as the standard, and a value greater than the upper quartile as the features that contribute significantly to the group were filtered.

### Differential Expressed Gene Analysis

Based on the gene expression data (counts) of the TCGA-BRCA dataset, we used the “DESeq2” package (Love et al., 2014) in R to analyze the DEGs between the two genomic subgroups. The screening criteria for DEGs is at *p* < 0.05 and absolute log2FC > 2.

### Prognostic Risk Model Construction and Analysis

The “coxph” function of the “survival” package in R was used to perform Cox analysis on samples and corresponding genes. In univariate Cox analysis, we considered the target gene as a factor that independently affected the prognosis for regression analysis and calculated the risk score and significance of each gene. The parameters used in univariate Cox analysis were:


coxph(formula= Surv(time,status)∼variable,data=clinical.data)

In the multivariate Cox analysis, we considered the target gene as a cofactor which related to other characteristics.

The parameters used in multivariate Cox analysis were:


coxph(formula= Surv(time,status)∼variable1+variable2+…+variable(i),data=clinical.data)

By analyzing the Cox regression coefficient of each gene, the sum of the Cox regression coefficient and the expression of the corresponding gene was used as the risk value to measure the risk of the sample. The risk score formula for each sample was calculated as follows:


Riskscore= -0.0076*FGF10(Exp)+0.0210*CSF3(Exp)+0.0288*NPY(Exp)

+0.0125*KLK3(Exp)+0.0158*SST(Exp)

-0.0250*XIRP2(Exp)+0.0275*C15orf43(Exp)

+0.0124*VCX3A(Exp)+0.0065*ISX(Exp)

+-0.0343*OR6T1(Exp)+0.0004*FDCSP(Exp)

Cox analysis was performed under the default parameters of the “coxph” function at the degree of significance of *p* < 0.05. The “glmnet” function of the “lars” package was used to perform LASSO analysis on the samples and corresponding genes. The parameters used in LASSO analysis were alpha = 1, nlambda = 100, and *p* < 0.05 was considered as the degree of significance.

### Copy Number Variation and Single-Nucleotide Polymorphism Analysis

Single-nucleotide polymorphism (SNV) analysis was based on the “maftools” package in R (Mayakonda et al., 2018). The default parameters were used to analyze the mutations of the TCGA-BRCA dataset. The statistics of mutation results are directly generated by the “oncplot” function of the “maftool” package. The analysis of CNV was performed using the GISTIC2.0 algorithm. The specific parameters used were set as follows: *-ta 0.1 -armpeel 1 -brlen 0.7 -cap 1.5 -conf 0.75 -td 0.1 -genegistic 1 -gcm extreme -js 4 -maxseg 2000 -qvt 0.25 -rx 0 -savegene 1*.

### Immune Infiltration Analysis

The tumor immune infiltration analysis was based on the gene expression data of TCGA-BRCA. The tumor immune cell ratio analysis of each sample was performed through the “Cibersort” software with the default parameters (Newman et al., 2015). Additionally, the ‘‘TIDE’’ software^2^ was used to analyze the difference in immune efficacy with the default parameters.

### Statistical Analysis

The unpaired Student’s *t*-test was used to analyze the comparison between two continuous variables and a normally distributed variable. Non-normally distributed variables were analyzed by the Mann–Whitney *U* test or Wilcoxon rank-sum test. To compare three or more groups, ANOVA and Kruskal–Wallis test were performed on the parametric method and the non-parametric method. The threshold of significance was at *P*-value < 0.05 or *P*-value < 0.01. Different significance levels were represented in different analyses.

## Results

### Genomic Variation Features to Classify the Cancer Genome Atlas Breast Cancer Samples Into Subgroups

**FIGURE 1:**
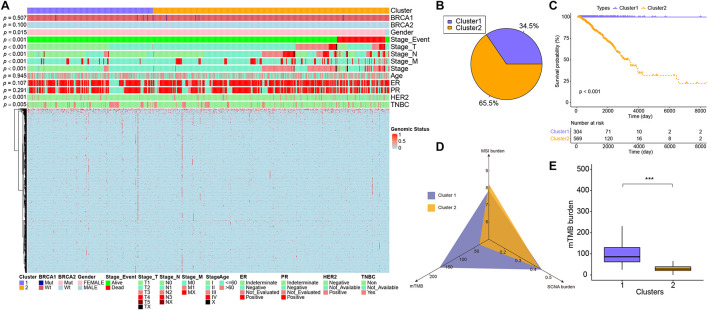
The difference in characteristics and genomic features between two subgroups. **(A)** The heatmap showed clusters of 1,274 genomic variation features. Sample annotations show the different clinical characteristics. **(B)** The sample proportion of the two subgroups. **(C)** Kaplan–Meier curve showed the overall survival in patients between cluster 1 and cluster 2 (*p* < 0.001). **(D)** The MSI burden, mTMB, and SCNA burden of each cluster. **(E)** The box plots showed the mTMB burden between cluster 1 and cluster 2. ***p < 0.001.

We characterized the three genome variants based on the MSI data, CNV data, and SNV data of the TCGA-BRCA dataset. After integrating the three genomic variation characteristics (a total of 1,536), the BRCA samples were separated into two genomic subgroups: cluster 1 and cluster 2 (Figure 1A). We found that cluster 2 basically gathered all the death cases, and was more inclined to have a higher-level stage, stage_M, stage_N, and stage_T on samples (*p* < 0.001), suggesting that the characteristics of genomic variation were significantly associated with tumor malignancy. Additionally, cluster 2 was also significantly associated with gender (*p* = 0.015), HER2 positive (*p* < 0.001), and triple-negative breast cancer (*p* = 0.005), but not BRCA1, BRCA2, ER, and PR (*p* > 0.05). Interestingly, there were 67 SCNA fragments among the 1,536 genomic characteristics, where 46 fragments were copy number amplifications, and 21 fragments were copy number deletions. The remaining features were all point mutations. In terms of point mutation types, samples with more gene mutations were clustered in cluster 1. These results suggest that, on the one hand, copy number amplification plays a more important role in the progression of breast cancer than copy number deletion. On the other hand, the malignancy of breast cancer is not contributed by all gene mutations. From the statistical point of view of the number of samples, the proportion of cluster 1 and cluster 2 samples was close to 7:13 (Figure 1B).

### Differences in Microsatellite Instability Burden, Somatic Copy Number Alteration Burden, and modified TMB Between the Two Subgroups

**FIGURE 2:**
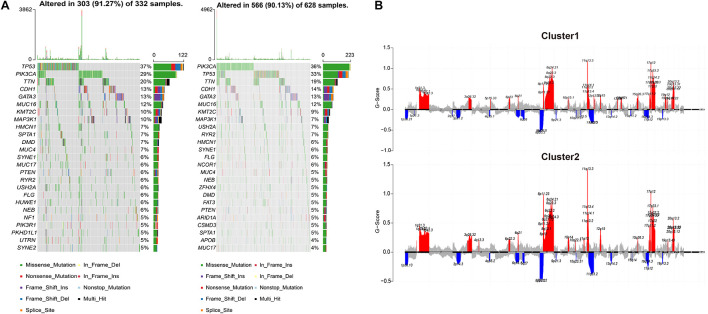
The differences in CNV and copy number variation between the two subgroups. **(A)** The oncoprint plots indicate the top 25 mutated genes in both cluster 1 and cluster 2 samples. Different colors stand for different mutation types. **(B)** Cumulative CNV regions for cluster 1 and cluster 2. Deletions are represented in blue color, and amplifications are represented in red color.

Next, the relevant indicators between subgroups were analyzed. In terms of survival differences between subgroups, the patients’ outcomes of cluster 1 were significantly better than that of cluster 2 (log-rank, *p* < 0.001, Figure 1C). In terms of the three traditional genomic variation measures of MSI burden, SCNA burden, and mTMB, these three genomic features may have a distinct impact on the biology of tumors (Figure 1D). Our model stratified all tumor samples into two clusters, where cluster 1 appeared to be genomically unstable. Specifically, cluster 1 and cluster 2 had no significant differences in MSI burden and SCNA burden (Supplementary Figure 2), but the mTMB burden in cluster 2 was relatively lower than cluster 1 (Figure 1E). To clarify the differences between the two subgroups at the genomic level in detail, the mutation landscape of the two subgroups are shown in Figure 2A. It can be seen that the mutation load of cluster 2 was less than that of cluster 1, which is consistent with the result in Figure 1E. Notably, most of the mutant genes were shared between the two subgroups, and the proportion of PIK3CA gene mutation in cluster 2 was greater than in cluster 1, and TP53 was mostly mutated in cluster 1. The CNV landscapes of the two subgroups are shown in Figure 2B, respectively. There was a statistically non-significant difference between cluster 1 and cluster 2, as reflected in Supplementary Figure 2B.

### Immune Landscape Between Two Subgroups

To investigate the immune cell infiltrations between the two subgroups, we performed CIBERSORT analysis to identify the abundance of 22 immune cell types (Supplementary Figure 3A), Only the ratio of Mast cells resting showed an increasing tendency (*p* = 0.019) in cluster 2 than in cluster 1 but not in other cell types (Supplementary Figure 3B). In addition, we counted the expression level of 18 immune checkpoints in the two subgroups, including *CD274 (PD-1), PDCD1 (PD-L1), CTLA4, LAG3, HAVCR2 (TIM-3), C10orf54 (VISTA), BTLA, CD200, CD200R1, CD276, CD40, CD40LG, CD80, CEACAM1, ICOS, IDO1, PDCD1LG2*, and *TIGIT*. However, there was no significant difference between cluster 1 and cluster 2 of all 18 immune checkpoints (Supplementary Figure 4). The immune dysfunction and exclusion levels were then calculated by TIDE, as shown in Supplementary Figure 5, there was no difference between cluster 1 and cluster 2 in the TIDE score.

### Construction of the Genomic Variation-Related Prognostic Risk Model (GVRM)

**FIGURE 3:**
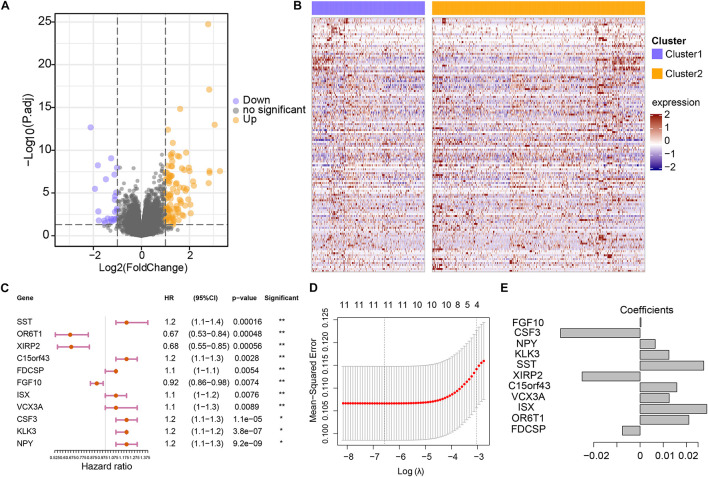
Construction of the genomic variation-related prognostic risk model. **(A)** The volcano plot indicates the association between log2 fold change and *P*-value in DEGs between cluster 1 and cluster 2. **(B)** The abundance of differentially expressed genes between cluster 1 and cluster 2. The row names of the heatmaps indicate the 130 DEGs, while the columns of the heatmaps indicate the samples. **(C)** Univariate analyses of 11 significant genes with overall survival (*p < 0.05 and **p < 0.01). **(D)** The partial likelihood deviation (λ) under each logarithm in the LASSO Cox regression model. **(E)** The coefficients of each independent variable in the LASSO Cox regression model.

To further assess the differences in gene expression levels between the two subgroups, a total of 130 DEGs were acquired by DESeq2 with the cut-off of *P* < 0.01 and absolute log2FC > 2 (Supplementary Table 2 and Figures 3A,B), where 100 genes were up-regulated in cluster 2, and 30 genes were down-regulated. Subsequently, these 130 genes were projected into univariate Cox analysis (Supplementary Table 3). As a result, 11 genes were identified to be significantly associated with survival (*P* < 0.01), in which *FGF10, OR6T1, and XIRP2* were considered to be favorable factors, and SST, *XIRP2, C15orf43, FDCSP, ISX, VCX3A, CSF3, KLK3*, and *NPY* were risk factors (Figure 3C). After 1,000 iterations of LASSO-penalized multivariate modeling, an 11 coefficient-based-risk model called GVRM was constructed (Figures 3D,E and Supplementary Table 4).

### Prognostic Performance Analysis of Risk Model and External Datasets Verification

**FIGURE 4:**
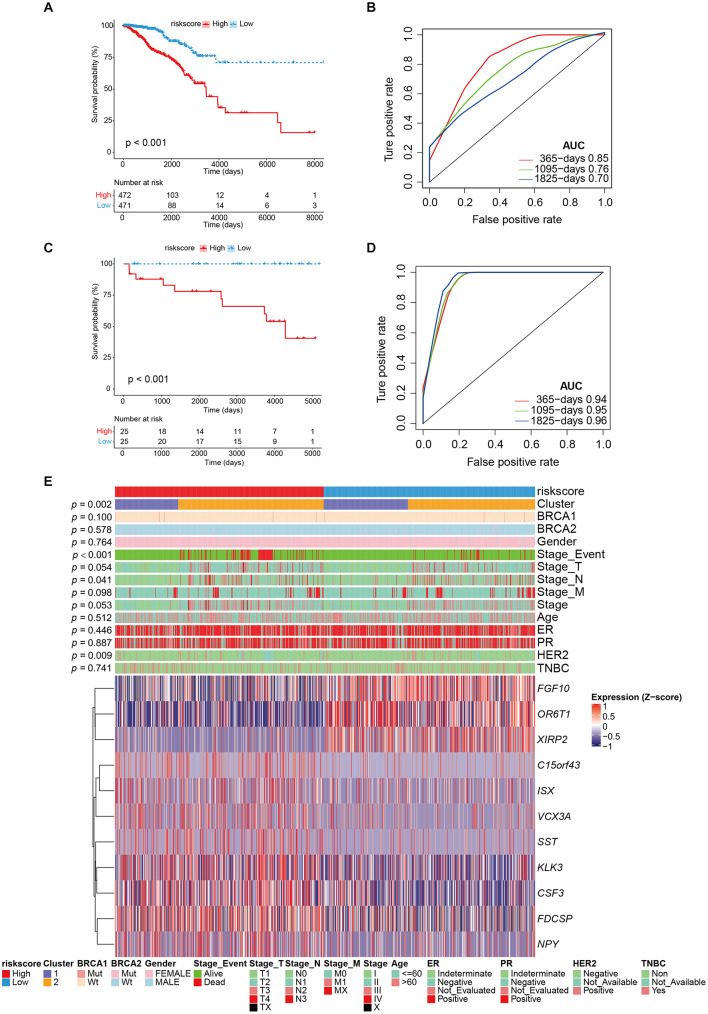
The performance of risk model and external validation. **(A)** Kaplan–Meier curve showed the overall survival in patients with high risk and low risk (*p* < 0.001). **(B)** The prognostic values of GVRM in 1-, 3-, and 5-year OS with AUC = 0.85, 0.76, and 0.7, respectively. **(C)** Kaplan–Meier curve showed the overall survival in the validation group (ICGC-BRCA-KR) with high risk and low risk (*p* < 0.001). **(D)** The prognostic values of GVRM in validation group in 1-, 3-, and 5-year OS with AUC = 0.94, 0.95, and 0.96, respectively. **(E)** The abundance of 11 significant genes (involved in GVRM) with clinical features in the training group (TCGA-BRCA).

Patients were divided into high- and low-risk groups according to the median value of risk score. There was a significant difference in OS between high- and low-risk groups (*p* < 0.001; Figure 4A), and the AUC values in 1-, 3-, and 5-year were all greater than 70% (Figure 4B), which suggests a promising prognostic predictive ability in the training (TCGA-BRCA) dataset. Remarkably, in the validation cohort (ICGC_BRCA_KR), this risk model also indicated a significant difference between the high and low-risk groups in patient outcomes (Figure 4C), the AUC values in 1-, 3-, and 5-year were all greater than 90% (Figure 4D). From the distribution of clinical characteristics in the high-risk and low-risk groups in the TCGA dataset, the occurrence of death events was relatively enriched in the high-risk group (*p* < 0.001), as well as in high stage_T (*p* = 0.047), and stage_N (*p* = 0.046). For the well-known breast cancer biomarkers, only HER2 status was statistically significantly associated with high risk (*p* = 0.010), but not others (BRCA1, BRCA2, ER, PR, *p* > 0.05). Particularly, cluster 2 was more enriched in the high-risk group, which is consistent with the observation that cluster 2, the genetically stable group, was significantly associated with poor prognosis (Figures 1C, 4E).

### Evaluation of Predictive Efficiency and Stability on GVRM

**FIGURE 5:**
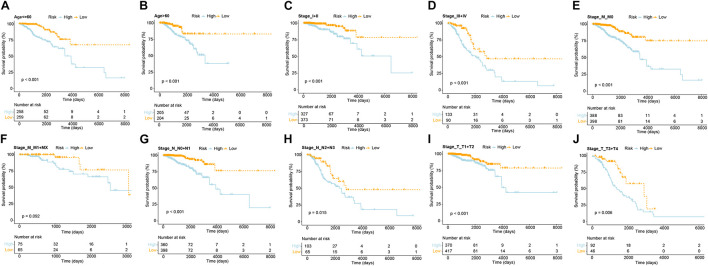
The efficiency and stability evaluation of GVRM. **(A,B)** Kaplan–Meier curve showed the overall survival in patients with high and low risk in age subgroups (*p* < 0.0001 and *p* < 0.0001, respectively). **(C,D)** Kaplan–Meier curve showed the overall survival in patients with high and low risk in stage subgroups (*p* < 0.001 and *p* < 0.001, respectively). **(E,F)** Kaplan–Meier curve showed the overall survival in patients with high and low risk in stage_M subgroups (*p* < 0.001 and *p* = 0.092, respectively). **(G,H)** Kaplan–Meier curve showed the overall survival in patients with high and low risk in stage_N subgroups (*p* < 0.001 and *p* = 0.015, respectively). **(I,J)** Kaplan–Meier curve showed the overall survival in patients with high and low risk in stage_T subgroups (*p* < 0.001 and *p* = 0.006, respectively).

The indicative clinical characteristics of the samples, including age, stages, stage_T, stage_M, and stage_N were used to evaluate the efficiency and stability of GVRM. We found that the risk model has significant differences between high- and low-risk samples in age, stage, stage_N, stage_M0, and stage_T (*p* < 0.05; Figures 5A–J). The results suggested the high efficiency and stability of the GVRM. We next counted the infiltration of 22 immune cells between high- and low-risk samples. A higher proportion of gamma delta T cells was detected to be up-regulated in high-risk patients (*p* = 0.031), while activated NK cells were detected to be up-regulated in low-risk patients (*p* = 0.039; Supplementary Figure 6).

**FIGURE 6:**
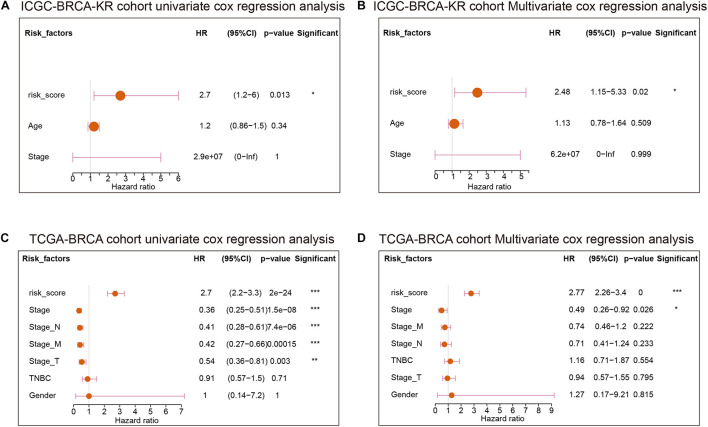
The Cox regression of clinical characteristics and risk score of GVRM. **(A,B)** The univariate Cox regression analysis and multivariate Cox regression of clinical characteristics and risk score of GVRM in the ICGC-BCRA-KR cohort, respectively. **(C,D)** The univariate Cox regression analysis and multivariate Cox regression of clinical characteristics and risk score of GVRM in TCGA-BRCA cohort, respectively. *p < 0.05, **p < 0.01 and ***p < 0.001.

**FIGURE 7:**
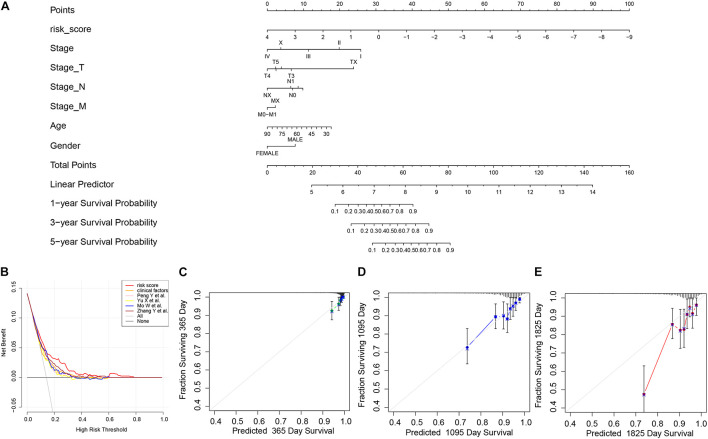
The nomogram of GVRM and its clinical benefit. **(A)** Nomogram for predicting 1-, 3-, and 5-year survival probability of BRCA patients in the training group. The total score of clinical characteristics, as well as risk score for each patient, is located on the “Total points” axis, which corresponds to the survival probabilities plotted on the three axes below. **(B)** Decision curve for the risk model, clinical factors, and other existing prognostic models. **(C–E)** Calibration curves for nomogram in 1-, 3-, and 5-year, respectively.

We thereafter analyzed the prognostic effects of risk score for different clinical characteristics in both TCGA and ICGC-BRCA-KR datasets. Univariate Cox regression and multivariate Cox regression demonstrated that the risk score was an independent prognostic factor in breast cancer (Figure 6). We then generated a nomogram to combine the clinical variables as well as risk scores to evaluate the clinical benefits, then the 1-, 3-, and 5-year survival probabilities were projected to the final sum of the scores (Figure 7A). In addition, decision curves for the nomogram and risk score prediction model are shown in Figure 7B; it can be observed that our risk model performs better than the other existing models (Mo et al., 2020; Peng et al., 2021; Yu et al., 2021; Zhang et al., 2021). The calibration plot of the nomogram agreed with the predictions of 3-, 5-, and 10-year OS, respectively (Figures 7C–E).

## Discussion

In this study, we characterized the molecular features of two genomic clusters divided by integrated analysis of three genomic variants including MSI, CNV, and SNV in breast cancers. We did not find the genomic variant-based clusters associated with specific immune landscape or response of immune checkpoints. TMB, the number of somatic mutations per megabase (Mb) of the genome, has been recognized as a potential biomarker of the immune response. Several studies reported higher response rates and improved PFS in patients in breast cancer with high TMB, who received PD-1/PD-L1 immunotherapy (Barroso-Sousa et al., 2020; Karn et al., 2020; Garrido-Castro et al., 2021). While TMB was significantly higher in cluster 1 in our study, the TIDE score between cluster 1 and cluster 2 was not significantly different, suggesting there might be other molecular mechanisms involved in the responsiveness of PD-1 blockade. Of note, higher CNV was inversely correlated with clinical benefit from ICB (Davoli et al., 2017). The higher CNV level in cluster 1, though not significantly different from cluster 2, may mitigate the prediction efficacy of immune response.

The association between genetic variants and breast cancer prognosis has been explored in terms of CNV, TMB, and MSI. CNV has been found to strongly associate with prognostic gene expression signatures in breast cancer (Horlings et al., 2010). Fatima et al. revealed that all MammaPrint genes, which were selected to predict breast cancer prognosis, had recurrent amplifications and deletions. The concordance between CNV-based genomic alterations and expression profiling of these genes indicates that copy number alterations play an important factor in tumor progression (Fatima et al., 2017). Although the frequency of MSI-H breast cancer is reportedly low ranging from 0.5 to 1% (Hause et al., 2016; Bonneville et al., 2017), high discordance between MSI-high and Mismatched Repair (MMR) proteins loss indicate the complexity of intra-tumor heterogeneity in patients with breast cancer (Fusco et al., 2018). MMR deficiency was found to be a prognostic marker in breast cancer (Fusco et al., 2018). TMB is an emerging biomarker for immunotherapy response and immune-related survival in breast cancer (Thomas et al., 2018). These findings suggest genetic variants-related gene signatures might be used as a potential tool for predicting breast cancer prognosis. In our study, we constructed the prognostic model based on the DEGs between the two clusters derived from integrated analysis of CNV, MSI, and TMB. We validated the prediction accuracy of this prognostic model in the ICGC breast cancer cohort. This provides a robust set of gene expression signatures and avoids the need for the laborious direct measurement of genetic variants at the DNA level.

In this study, we found that our prediction model is superior to previous prognostic models of breast cancer. Of note, these prognostic models were constructed by immune-related genes or immune infiltration scores (Peng et al., 2021; Yu et al., 2021; Zhang et al., 2021) and the CNV-related gene expression model (Mo et al., 2020). Our findings suggested that genomic variants-based risk stratification may be more relevant to breast cancer prognosis compared with immune-related gene models. Our risk model showed better performance of prognosis prediction than the CNV-based gene expression model by Mo et al., suggesting that integrated analysis of CNV, TMB, and MSI may capture more accurate determinants of prognosis in breast cancer.

Among the 11-gene signature, several genes were involved in breast cancer growth. High expression of FGF10 has been reported in about 10% of breast cancer and correlated with tumor progression (Theodorou et al., 2004). Therapeutic targeting of FGFs and their receptors (FGFRs) is currently under active research. CSF3 (G-CSF) is a cytokine stimulating mobilization of hematopoietic stem cells from bone marrow and promoting differentiation of neutrophil precursors. High expression of G-CSF secreted from breast cancer cells promotes tumor-associated macrophages into the inflammatory phenotype, leading to tumor growth and poor survival (Hollmén et al., 2015). KLK3 (PSA) is an original androgen receptor-governed prostate kallikreins. As androgen receptor pathway activation has been found to promote a subset of breast cancer, the role of KLK3 involved in tumor progression may need further investigation (Thorek et al., 2019). Neuropeptide Y has been found to promote proliferation and migration in breast cancer cells (Medeiros et al., 2012).

In summary, we performed an integrated analysis of three genetic variants including CNV, MSI, and TMB, which generated two distinct subgroups. We characterized the association between the two groups in terms of prognosis, mutation, genetic variants, and immune landscape. We further developed an 11-gene signature based on the DEGs between these two subgroups. We constructed this GVRM and validated its prognostic prediction in an independent cohort (ICGC-BRCA-KR). Furthermore, we demonstrated that the performance efficiency was superior to previous published prognostic models. Consequently, we developed a nomogram to help clinicians estimate prognosis at the individual level.

## Data Availability Statement

The datasets presented in this study can be found in online repositories. The names of the repository/repositories and accession number(s) can be found in the article/Supplementary Material.

## Author Contributions

YC and W-KH conceived the study design. XJ and JY performed RNA-seq analyses. XJ, JY, and CC performed cluster and classification analyses. XJ drafted the manuscript. JY, CC, YC, and W-KH participated in revision of the manuscript. All authors read and approved this final manuscript.

## Conflict of Interest

The authors declare that the research was conducted in the absence of any commercial or financial relationships that could be construed as a potential conflict of interest.

## Publisher’s Note

All claims expressed in this article are solely those of the authors and do not necessarily represent those of their affiliated organizations, or those of the publisher, the editors and the reviewers. Any product that may be evaluated in this article, or claim that may be made by its manufacturer, is not guaranteed or endorsed by the publisher.

## Footnotes

1
http://xena.ucsc.edu/

2
https://github.com/liulab-dfci/TIDEpy

## Supplementary Material

Supplementary Table 1 The clinical characteristics in the TCGA BRCA cohort (*n* = 1079).

Supplementary Table 2 The DEGs between two genomic variation subgroups (top20, cluster 1 vs. cluster 2).

Supplementary Table 3 The univariate Cox results of 130 DEGs.

Supplementary Table 4 The coefficients of 11 genes involved in GVRM.

## References

[B1] Adams S. Gatti-Mays M. E. Kalinsky K. Korde L. A. Sharon E. Amiri-Kordestani L. (2019). Current landscape of immunotherapy in breast cancer: a review. *JAMA Oncol.* 5 1205–1214. 10.1001/jamaoncol.2018.7147 30973611 PMC8452050

[B2] Barroso-Sousa R. Keenan T. E. Pernas S. Exman P. Jain E. Garrido-Castro A. C. (2020). Tumor mutational burden and PTEN alterations as molecular correlates of response to PD-1/L1 blockade in metastatic triple-negative breast cancer. *Clin. Cancer Res.* 26 2565–2572. 10.1158/1078-0432.CCR-19-3507 32019858 PMC7269810

[B3] Bates J. P. Derakhshandeh R. Jones L. Webb T. J. (2018). Mechanisms of immune evasion in breast cancer. *BMC Cancer* 18:556. 10.1186/s12885-018-4441-3 29751789 PMC5948714

[B4] Bonneville R. Krook M. A. Kautto E. A. Miya J. Wing M. R. Chen H. Z. (2017). Landscape of microsatellite instability across 39 cancer types. *JCO Precis Oncol.* 2017 1–15. 10.1200/PO.17.00073 29850653 PMC5972025

[B5] Bonotto M. Gerratana L. Poletto E. Driol P. Giangreco M. Russo S. (2014). Measures of outcome in metastatic breast cancer: insights from a real-world scenario. *Oncologist* 19 608–615. 10.1634/theoncologist.2014-0002 24794159 PMC4041678

[B6] Ciriello G. Miller M. L. Aksoy B. A. Senbabaoglu Y. Schultz N. Sander C. (2013). Emerging landscape of oncogenic signatures across human cancers. *Nat. Genet.* 45 1127–1133. 10.1038/ng.2762 24071851 PMC4320046

[B7] Cortes-Ciriano I. Lee S. Park W. Y. Kim T. M. Park P. J. (2017). A molecular portrait of microsatellite instability across multiple cancers. *Nat Commun.* 8:15180. 10.1038/ncomms15180 28585546 PMC5467167

[B8] Cristescu R. Mogg R. Ayers M. Albright A. Murphy E. Yearley J. (2018). Pan-tumor genomic biomarkers for PD-1 checkpoint blockade-based immunotherapy. *Science* 362 eaar3593. 10.1126/science.aar3593 30309915 PMC6718162

[B9] Davoli T. Uno H. Wooten E. C. Elledge S. J. (2017). Tumor aneuploidy correlates with markers of immune evasion and with reduced response to immunotherapy. *Science* 355:eaaf8399. 10.1126/science.aaf8399 28104840 PMC5592794

[B10] DeSantis C. E. Ma J. Gaudet M. M. Newman L. A. Miller K. D. Goding Sauer A. (2019). Breast cancer statistics, 2019. *CA Cancer J. Clin.* 69 438–451. 10.3322/caac.21583 31577379

[B11] Fatima A. Tariq F. Malik M. F. A. Qasim M. Haq F. (2017). Copy number profiling of mammaprint genes reveals association with the prognosis of breast cancer patients. *J. Breast Cancer* 20 246–253. 10.4048/jbc.2017.20.3.246 28970850 PMC5620439

[B12] Fusco N. Lopez G. Corti C. Pesenti C. Colapietro P. Ercoli G. (2018). Mismatch repair protein loss as a prognostic and predictive biomarker in breast cancers regardless of microsatellite instability. *JNCI Cancer Spectr.* 2:ky056. 10.1093/jncics/pky056 31360876 PMC6649738

[B13] Garrido-Castro A. C. Spurr L. F. Hughes M. E. Li Y. Y. Cherniack A. D. Kumari P. (2021). Genomic characterization of de novo metastatic breast cancer. *Clin. Cancer Res.* 27 1105–1118. 10.1158/1078-0432.CCR-20-1720 33293374 PMC7887078

[B14] Hause R. J. Pritchard C. C. Shendure J. Salipante S. J. (2016). Classification and characterization of microsatellite instability across 18 cancer types. *Nat. Med.* 22 1342–1350. 10.1038/nm.4191 27694933

[B15] Hollmén M. Karaman S. Schwager S. Lisibach A. Christiansen A. J. Maksimow M. (2015). G-CSF regulates macrophage phenotype and associates with poor overall survival in human triple-negative breast cancer. *Oncoimmunology* 5:e1115177. 10.1080/2162402X.2015.1115177 27141367 PMC4839343

[B16] Horlings H. M. Lai C. Nuyten D. S. Halfwerk H. Kristel P. Van Beers E. (2010). Integration of DNA copy number alterations and prognostic gene expression signatures in breast cancer patients. *Clin. Cancer Res.* 16 651–663. 10.1158/1078-0432.CCR-09-0709 20068109

[B17] Kandoth C. Mclellan M. D. Vandin F. Ye K. Niu B. Lu C. (2013). Mutational landscape and significance across 12 major cancer types. *Nature* 502 333–339. 10.1038/nature12634 24132290 PMC3927368

[B18] Karn T. Denkert C. Weber K. E. Holtrich U. Hanusch C. Sinn B. V. (2020). Tumor mutational burden and immune infiltration as independent predictors of response to neoadjuvant immune checkpoint inhibition in early TNBC in GeparNuevo. *Ann. Oncol.* 31 1216–1222. 10.1016/j.annonc.2020.05.015 32461104

[B19] Le D. T. Durham J. N. Smith K. N. Wang H. Bartlett B. R. Aulakh L. K. (2017). Mismatch repair deficiency predicts response of solid tumors to PD-1 blockade. *Science* 357 409–413. 10.1126/science.aan6733 28596308 PMC5576142

[B20] Le D. T. Uram J. N. Wang H. Bartlett B. R. Kemberling H. Eyring A. D. (2015). PD-1 blockade in tumors with mismatch-repair deficiency. *N. Engl. J. Med.* 372 2509–2520. 10.1056/NEJMoa1500596 26028255 PMC4481136

[B21] Li L. Feng Q. Wang X. (2020). PreMSIm: an r package for predicting microsatellite instability from the expression profiling of a gene panel in cancer. *Comput. Struct. Biotechnol. J.* 18 668–675. 10.1016/j.csbj.2020.03.007 32257050 PMC7113609

[B22] Love M. I. Huber W. Anders S. (2014). Moderated estimation of fold change and dispersion for RNA-seq data with DESeq2. *Genome Biol.* 15:550. 10.1186/s13059-014-0550-8 25516281 PMC4302049

[B23] Mayakonda A. Lin D. C. Assenov Y. Plass C. Koeffler H. P. (2018). Maftools: efficient and comprehensive analysis of somatic variants in cancer. *Genome Res.* 28 1747–1756. 10.1101/gr.239244.118 30341162 PMC6211645

[B24] Medeiros P. J. Al-Khazraji B. K. Novielli N. M. Postovit L. M. Chambers A. F. Jackson D. N. (2012). Neuropeptide Y stimulates proliferation and migration in the 4T1 breast cancer cell line. *Int. J. Cancer.* 131 276–286. 10.1002/ijc.26350 21823118

[B25] Mermel C. H. Schumacher S. E. Hill B. Meyerson M. L. Beroukhim R. Getz G. (2011). GISTIC2.0 facilitates sensitive and confident localization of the targets of focal somatic copy-number alteration in human cancers. *Genome Biol.* 12:R41. 10.1186/gb-2011-12-4-r41 21527027 PMC3218867

[B26] Mo Q. Wang S. Seshan V. E. Olshen A. B. Schultz N. Sander C. (2013). Pattern discovery and cancer gene identification in integrated cancer genomic data. *Proc. Natl. Acad. Sci. U.S.A.* 110 4245–4250. 10.1073/pnas.1208949110 23431203 PMC3600490

[B27] Mo W. Ding Y. Zhao S. Zou D. Ding X. (2020). Identification of a 6-gene signature for the survival prediction of breast cancer patients based on integrated multi-omics data analysis. *PLoS One.* 15:e0241924. 10.1371/journal.pone.0241924 33170908 PMC7654770

[B28] Newman A. M. Liu C. L. Green M. R. Gentles A. J. Feng W. Xu Y. (2015). Robust enumeration of cell subsets from tissue expression profiles. *Nat. Methods* 12 453–457. 10.1038/nmeth.3337 25822800 PMC4739640

[B29] Passaro A. Stenzinger A. Peters S. (2020). Tumor mutational burden as a pan-cancer biomarker for immunotherapy: the limits and potential for convergence. *Cancer Cell* 38 624–625. 10.1016/j.ccell.2020.10.019 33171127

[B30] Peng Y. Yu H. Jin Y. Qu F. Ren H. Tang Z. (2021). Construction and validation of an immune infiltration-related gene signature for the prediction of prognosis and therapeutic response in breast cancer. *Front. Immunol.* 12:666137. 10.3389/fimmu.2021.666137 33986754 PMC8110914

[B31] Rizvi N. A. Hellmann M. D. Snyder A. Kvistborg P. Makarov V. Havel J. J. (2015). Cancer immunology. Mutational landscape determines sensitivity to PD-1 blockade in non-small cell lung cancer. *Science* 348 124–128. 10.1126/science.aaa1348 25765070 PMC4993154

[B32] Samstein R. M. Lee C. H. Shoushtari A. N. Hellmann M. D. Shen R. Janjigian Y. Y. (2019). Tumor mutational load predicts survival after immunotherapy across multiple cancer types. *Nat. Genet.* 51 202–206. 10.1038/s41588-018-0312-8 30643254 PMC6365097

[B33] Taylor A. M. Shih J. Ha G. Gao G. F. Zhang X. Berger A. C. (2018). Genomic and Functional approaches to understanding cancer aneuploidy. *Cancer Cell* 33 676–689.e3. 10.1016/j.ccell.2018.03.007 29622463 PMC6028190

[B34] Theodorou V. Boer M. Weigelt B. Jonkers J. Van Der Valk M. Hilkens J. (2004). Fgf10 is an oncogene activated by MMTV insertional mutagenesis in mouse mammary tumors and overexpressed in a subset of human breast carcinomas. *Oncogene* 23 6047–6055. 10.1038/sj.onc.1207816 15208658

[B35] Thomas A. Routh E. D. Pullikuth A. Jin G. Su J. Chou J. W. (2018). Tumor mutational burden is a determinant of immune-mediated survival in breast cancer. *Oncoimmunology* 7:e1490854. 10.1080/2162402X.2018.1490854 30386679 PMC6207420

[B36] Thorek D. L. J. Ku A. T. Mitsiades N. Veach D. Watson P. A. Metha D. (2019). Harnessing androgen receptor pathway activation for targeted alpha particle radioimmunotherapy of breast cancer. *Clin. Cancer Res.* 25 881–891. 10.1158/1078-0432.CCR-18-1521 30254080 PMC6524527

[B37] Yu X. Guo J. Zhou Q. Huang W. Xu C. Long X. (2021). A novel immune-related prognostic index for predicting breast cancer overall survival. *Breast Cancer* 28, 434–447. 10.1007/s12282-020-01175-z 33146847

[B38] Zhang Y. Di X. Chen G. Liu J. Zhang B. Feng L. (2021). An immune-related signature that to improve prognosis prediction of breast cancer. *Am. J. Cancer Res.* 11, 1267–1285. 33948357 PMC8085862

