# Supplementary material for: Integrated Analysis of Copy Number Variation, Microsatellite Instability, and Tumor Mutation Burden Identifies an 11-Gene Signature Predicting Survival in Breast Cancer
Source: Front Cell Dev Biol. 2021 Sep 28;9:721505. doi: 10.3389/fcell.2021.721505 (PMC8505672; doi:10.3389/fcell.2021.721505)
Supplement: Supplementary Table 1 — The clinical characteristics in the TCGA BRCA cohort (n = 1079). [file Table_1.DOCX]

Table S1. TCGA sample information

| **Clinical trials** | **type** | **sample number** |
| --- | --- | --- |
| Stage_T | T1 | 276 |
|  | T2 | 627 |
|  | T3 | 134 |
|  | T4 | 37 |
|  | T5 | 1 |
|  | T6 | 1 |
|  | TX | 3 |
| Stage_N | N0 | 512 |
|  | N1 | 355 |
|  | N2 | 117 |
|  | N3 | 76 |
|  | NX | 19 |
| Stage_M | M0 | 899 |
|  | M1 | 20 |
|  | MX | 160 |
| Stage | I | 182 |
|  | II | 617 |
|  | III | 247 |
|  | IV | 20 |
|  | X | 13 |
| ER | Indeterminate | 2 |
|  | Negative | 232 |
|  | Not_Evaluated | 48 |
|  | Positive | 797 |
| PR | Indeterminate | 4 |
|  | Negative | 337 |
|  | Not_Evaluated | 49 |
|  | Positive | 689 |
| HER | Negative | 936 |
|  | Not_Available | 65 |
|  | Positive | 78 |
| TNBC | Non | 876 |
|  | Not_Available | 9 |
|  | Yes | 194 |
| Age | <=60 | 590 |
|  | >60 | 489 |
