# Supplementary material for: Integrated Analysis of Copy Number Variation, Microsatellite Instability, and Tumor Mutation Burden Identifies an 11-Gene Signature Predicting Survival in Breast Cancer
Source: Front Cell Dev Biol. 2021 Sep 28;9:721505. doi: 10.3389/fcell.2021.721505 (PMC8505672; doi:10.3389/fcell.2021.721505)
Supplement: Supplementary Table 2 — The DEGs between two genomic variation subgroups (top20, cluster 1 vs. cluster 2). [file Table_2.DOCX]

Table S2. The DEGs between two genomic variation subgroups（top20, Cluster 2 vs Cluster 1）

| **Gene** | **baseMean** | **log2FoldChange** | **lfcSE** | **stat** | **pvalue** | **padj** | **type** |
| --- | --- | --- | --- | --- | --- | --- | --- |
| CHGB | 939.940667 | 2.77828513 | 0.24531033 | 11.3255936 | 9.80E-30 | 1.83E-25 | Up_in_Cluster2 |
| CSN1S1 | 20.3786661 | 2.82387299 | 0.294437 | 9.59075449 | 8.74E-22 | 8.14E-18 | Up_in_Cluster2 |
| LHFPL4 | 14.5309933 | 1.61425119 | 0.17948307 | 8.99389088 | 2.39E-19 | 1.48E-15 | Up_in_Cluster2 |
| SYT4 | 13.512059 | 3.05117091 | 0.35978049 | 8.48064587 | 2.24E-17 | 1.04E-13 | Up_in_Cluster2 |
| CSRP3 | 1.49469707 | -2.1143309 | 0.25255689 | -8.3717014 | 5.68E-17 | 2.12E-13 | Up_in_Cluster1 |
| SLC4A4 | 175.617476 | 1.101089 | 0.13305365 | 8.27552624 | 1.28E-16 | 3.97E-13 | Up_in_Cluster2 |
| KLK14 | 148.203279 | 1.30663474 | 0.16703832 | 7.82236511 | 5.18E-15 | 1.38E-11 | Up_in_Cluster2 |
| CA10 | 5.51673621 | 1.86265523 | 0.24934547 | 7.47017882 | 8.01E-14 | 1.86E-10 | Up_in_Cluster2 |
| ACE2 | 102.788375 | 1.29107328 | 0.17352746 | 7.440167 | 1.01E-13 | 2.08E-10 | Up_in_Cluster2 |
| CUX2 | 118.763687 | 1.21908293 | 0.16579091 | 7.35313484 | 1.94E-13 | 3.28E-10 | Up_in_Cluster2 |
| UCP1 | 28.9905066 | 1.5074951 | 0.2065813 | 7.29734534 | 2.94E-13 | 4.55E-10 | Up_in_Cluster2 |
| TRH | 393.252548 | 1.57469537 | 0.21683102 | 7.26231597 | 3.81E-13 | 5.45E-10 | Up_in_Cluster2 |
| FGF10 | 763.971897 | 1.24233484 | 0.17149865 | 7.24399184 | 4.36E-13 | 5.79E-10 | Up_in_Cluster2 |
| RFX4 | 3.43370122 | -1.2613144 | 0.17565207 | -7.1807545 | 6.93E-13 | 8.61E-10 | Up_in_Cluster1 |
| MPPED1 | 23.7765265 | 1.28615045 | 0.18218259 | 7.05967806 | 1.67E-12 | 1.94E-09 | Up_in_Cluster2 |
| XIRP2 | 4.08181011 | -1.8140194 | 0.2632027 | -6.8921004 | 5.50E-12 | 5.69E-09 | Up_in_Cluster1 |
| GPR115 | 56.6313599 | 1.0987481 | 0.15994574 | 6.86950538 | 6.44E-12 | 6.31E-09 | Up_in_Cluster2 |
| SEZ6 | 42.8232263 | 1.27858885 | 0.187077 | 6.83455941 | 8.23E-12 | 7.35E-09 | Up_in_Cluster2 |
| PCSK1 | 461.684904 | 1.23419896 | 0.18078517 | 6.82688169 | 8.68E-12 | 7.35E-09 | Up_in_Cluster2 |
| NPY | 5.49233011 | 1.97710726 | 0.2924129 | 6.76135444 | 1.37E-11 | 1.06E-08 | Up_in_Cluster2 |
