# Supplementary figures and images for: Integrated Analysis of Copy Number Variation, Microsatellite Instability, and Tumor Mutation Burden Identifies an 11-Gene Signature Predicting Survival in Breast Cancer
Source: Front Cell Dev Biol. 2021 Sep 28;9:721505. doi: 10.3389/fcell.2021.721505 (PMC8505672; doi:10.3389/fcell.2021.721505)

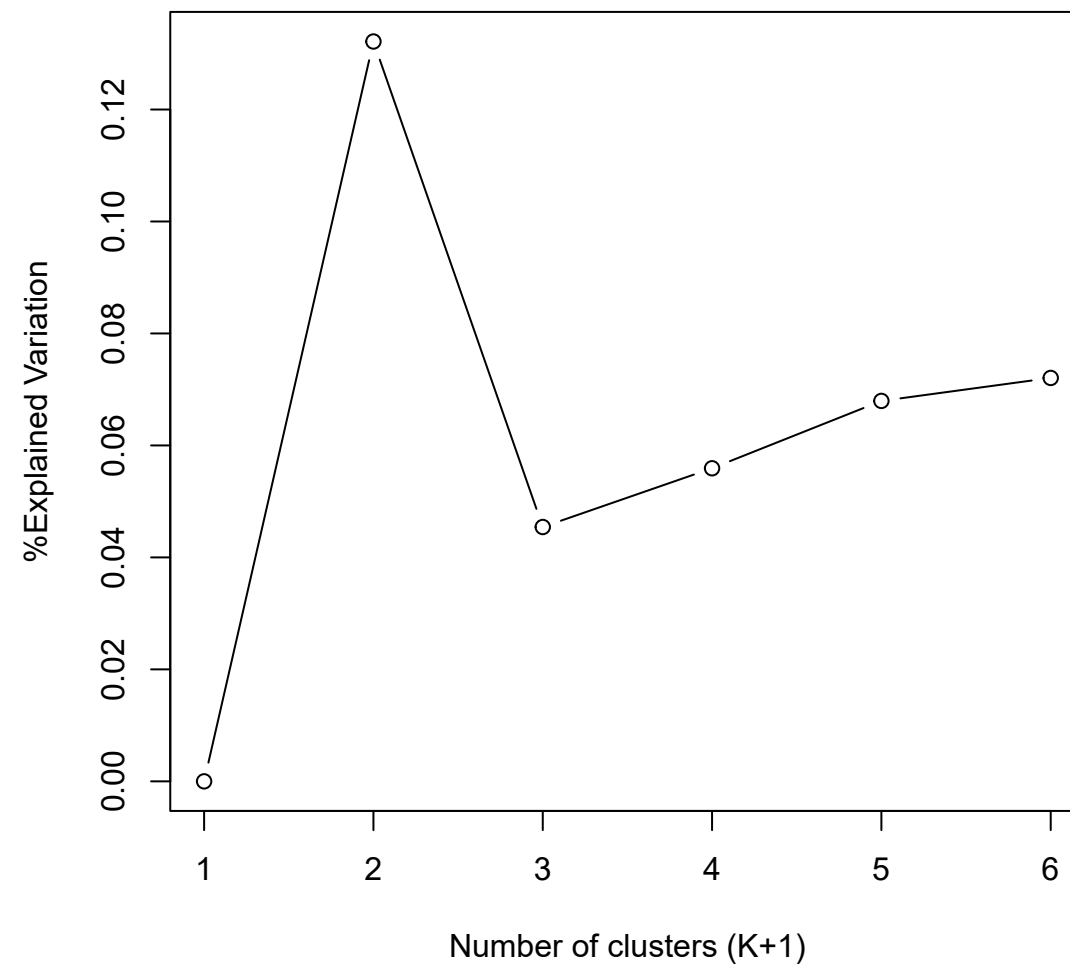

Supplement: Supplementary file 4 [file Data_Sheet_1.PDF]

A

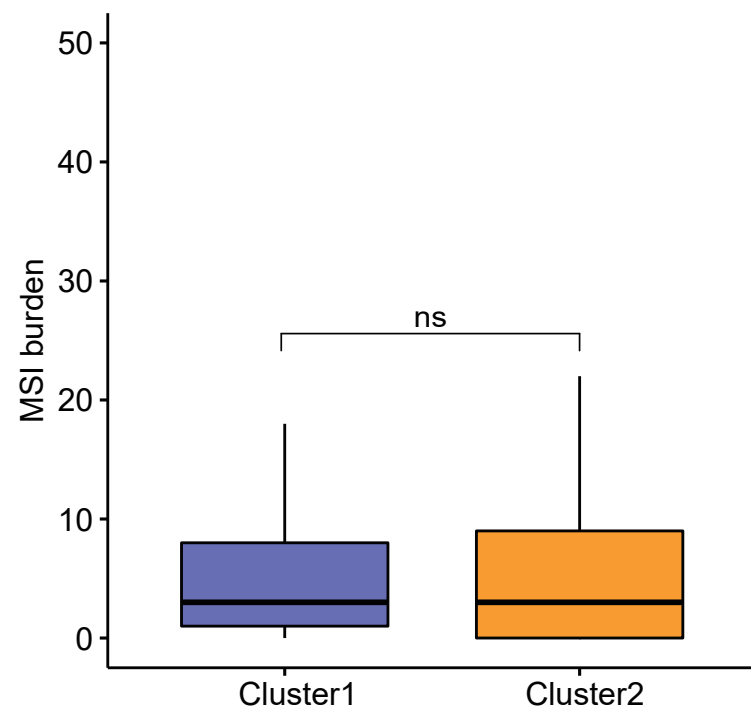

B

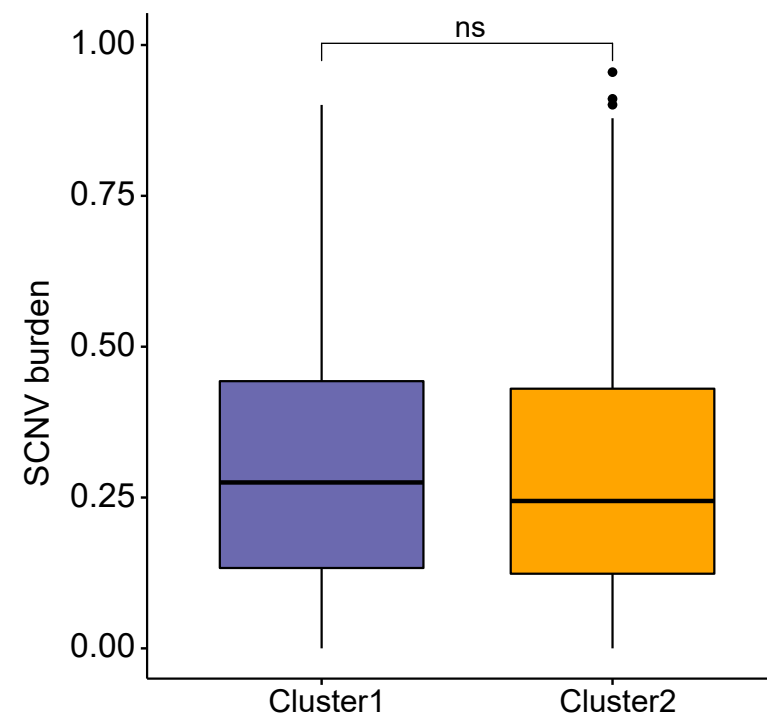

Supplement: Supplementary file 5 [file Data_Sheet_2.PDF]

A

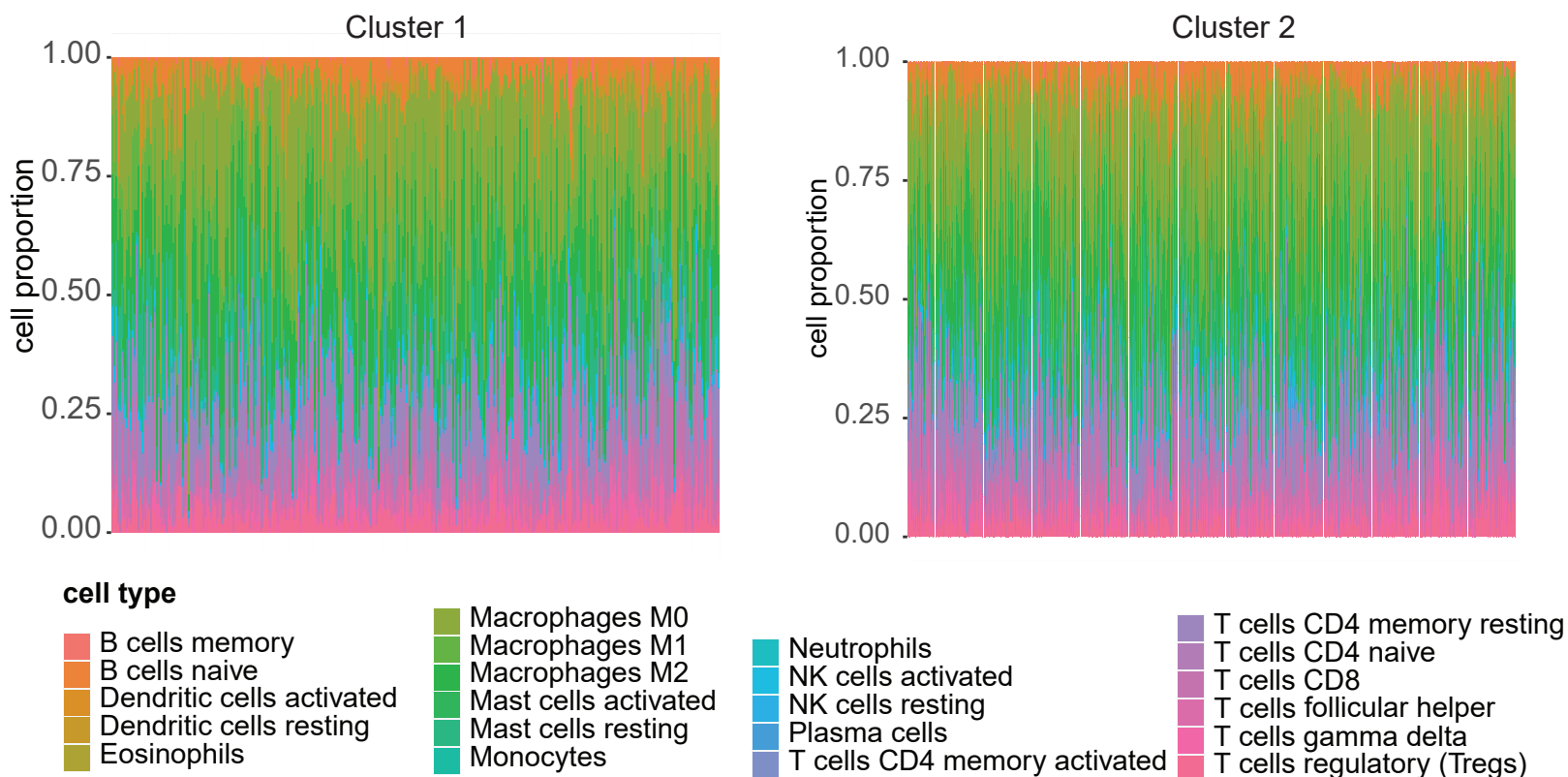

B

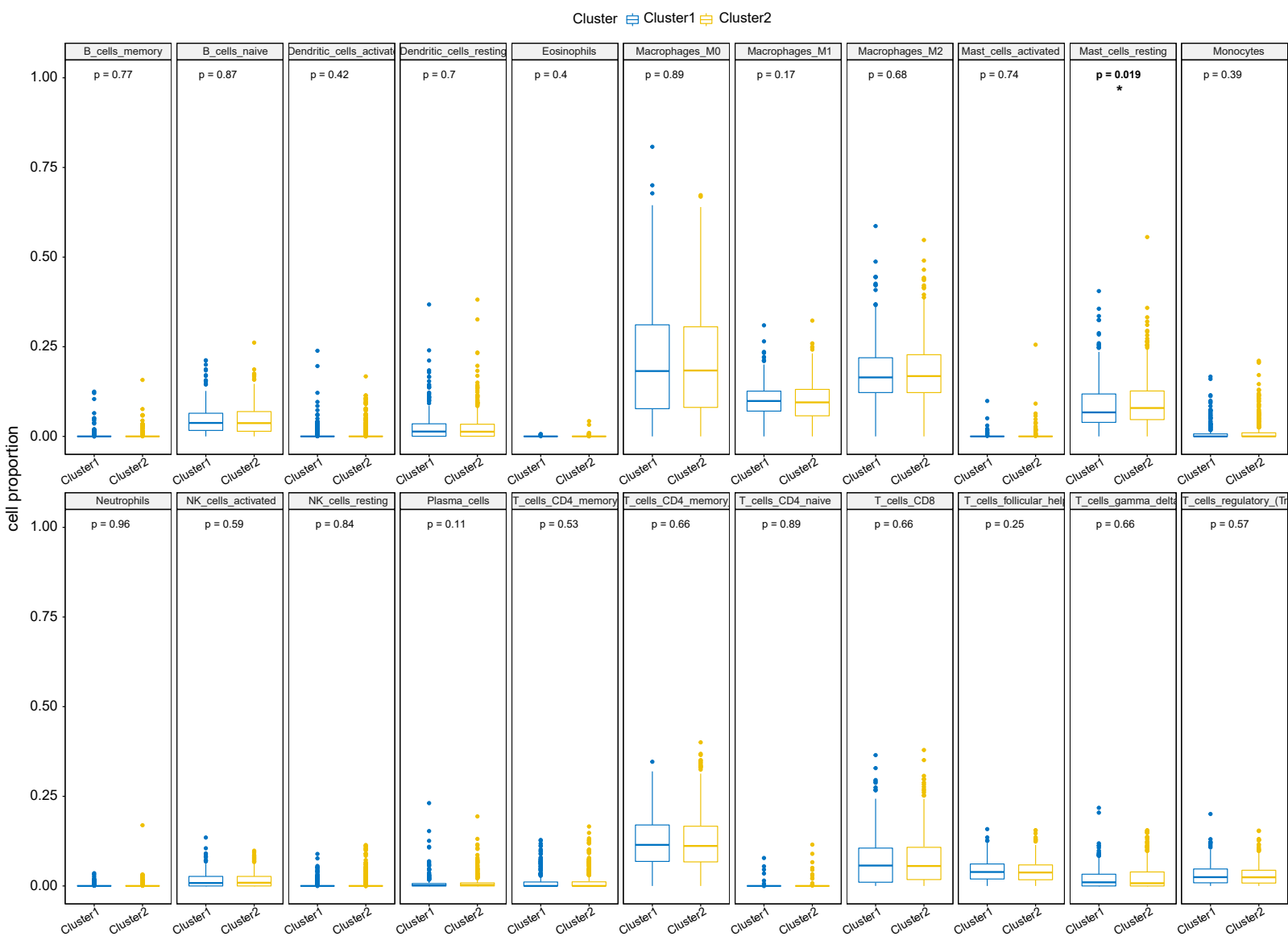

Supplement: Supplementary file 6 [file Data_Sheet_3.PDF]

Cluster 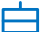 Cluster1 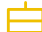 Cluster2

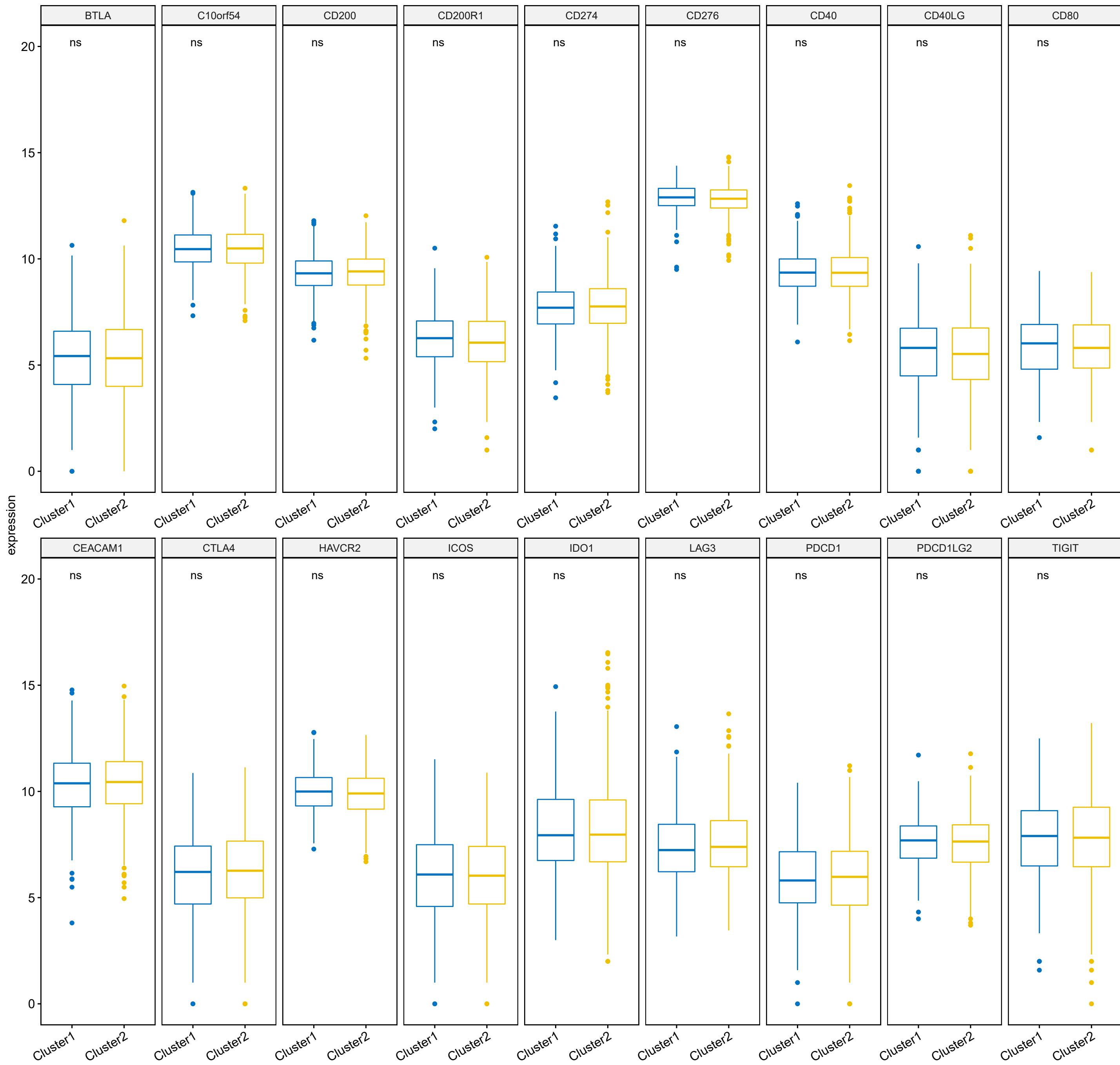

Supplement: Supplementary file 7 [file Data_Sheet_4.PDF]

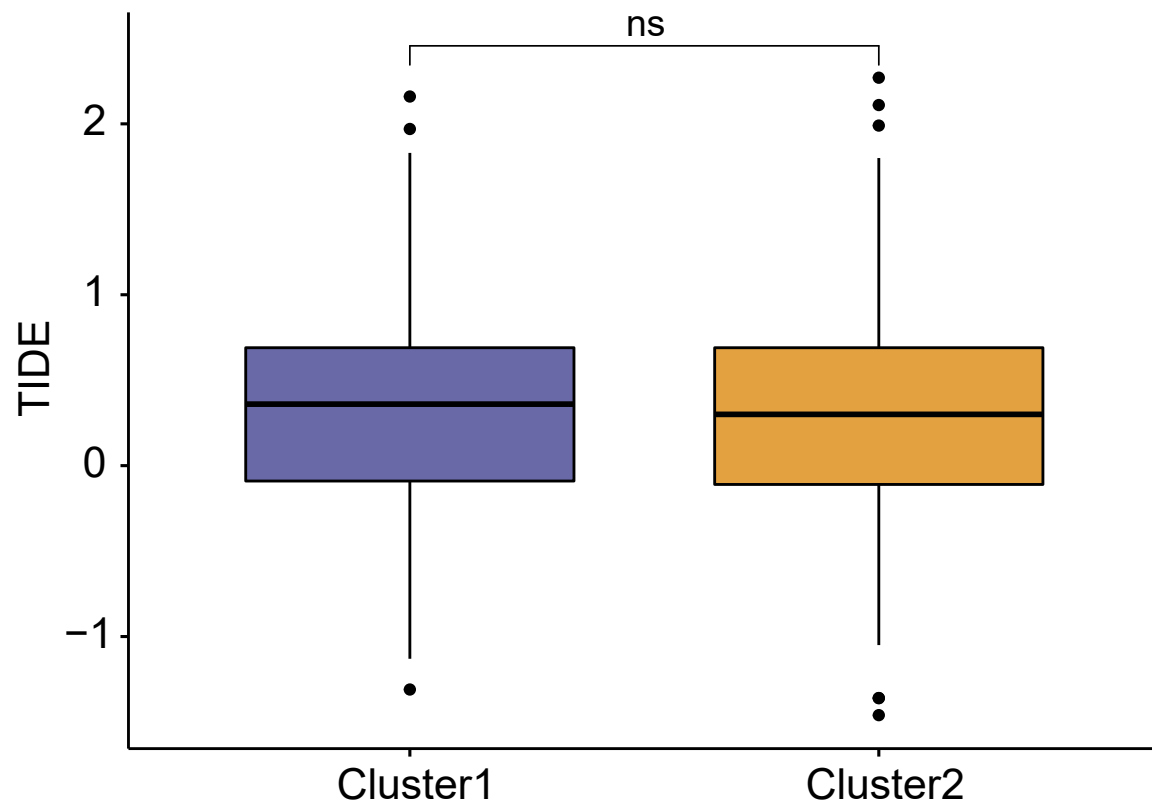

Supplement: Supplementary file 8 [file Data_Sheet_5.PDF]

Risk High Low

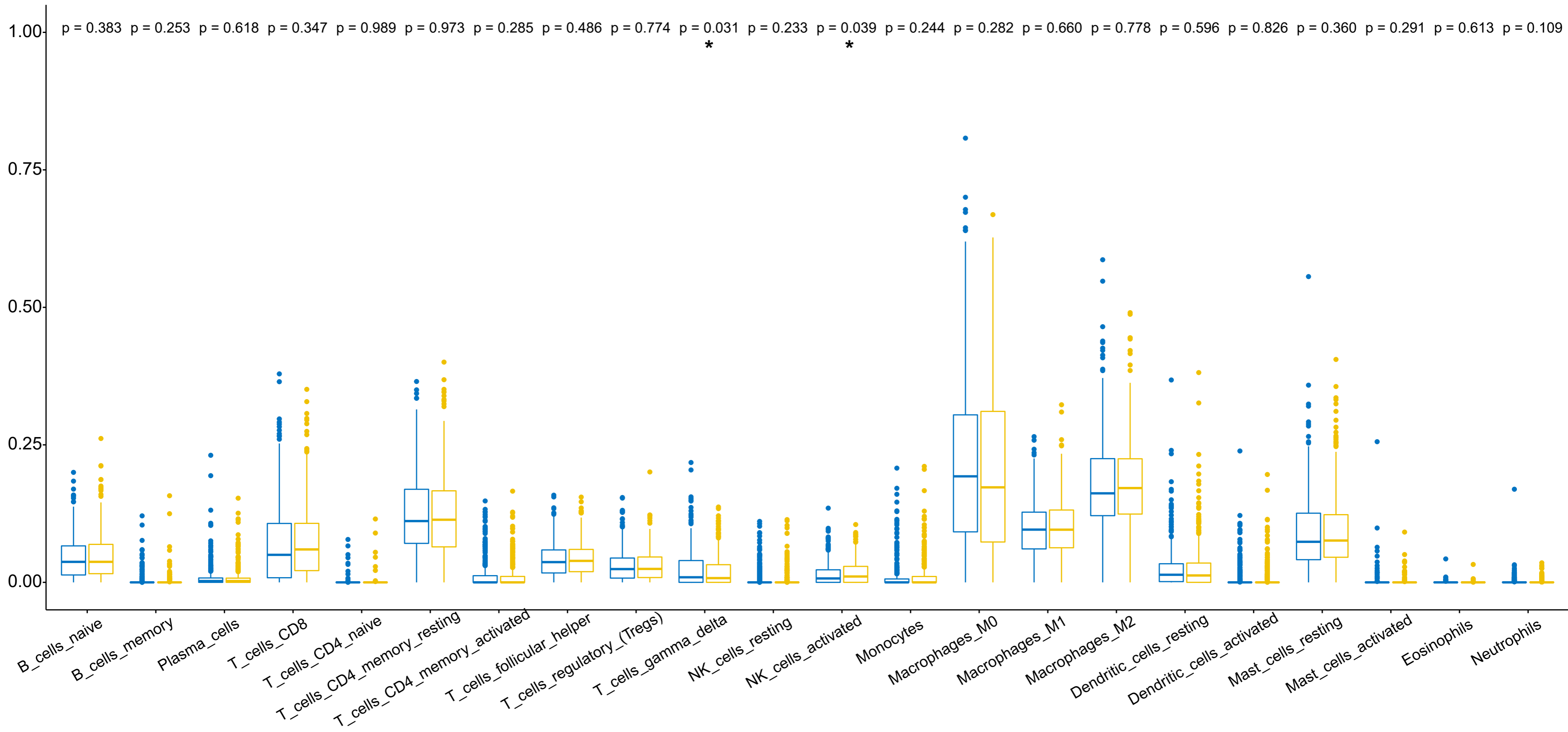

Supplement: Supplementary file 9 [file Data_Sheet_6.PDF]
